# Effectiveness of Platelet‐Rich Plasma in Anterior Cruciate Ligament Reconstruction: A Systematic Review of Randomized Controlled Trials

**DOI:** 10.1111/os.13279

**Published:** 2022-09-02

**Authors:** Yi Cao, Ye‐da Wan

**Affiliations:** ^1^ Radiology Department Tianjin Hospital Tianjin City China

**Keywords:** Anterior cruciate ligament, Clinical outcome, Image evaluation, Platelet‐rich plasma, Randomized controlled trials

## Abstract

This study aimed to identify the effectiveness of platelet‐rich plasma (PRP) for patients operated with anterior cruciate ligament reconstruction (ACLR).

Databases of PubMed, Embase, and CENTRAL were independently retrieved by two authors, for identifying the eligible randomized controlled trials (RCTs) comparing the clinical and imaging outcomes of ACL reconstructed patients augmented with or without PRP. The Cochrane Collaboration tool was utilized to assess the risk of bias of the included trials. We qualitatively synthesized the outcomes include the image evaluations on the healing of bone tunnels, graft remodeling, donor site healing and tunnel widening, and clinical evaluations on knee stability and function, pain symptom by visual analogue scale (VAS), inflammatory parameters and so on.

A total of 16 RCTs, including 1025 patients, were included for eligibility. Generally, the included studies were of low risk of bias, but the conducting of allocation concealment was not clearly described in many studies. Three imaging techniques, including MRI, CT and ultrasound, were selected in these trials. Significant improvement on graft remodeling, bone tunnel healing, harvest site healing and bone tunnel diameters were demonstrated in one of five (20.0%), three of five (60.0%), two of four (50.0%) and one of five (20.0%) studies respectively, for PRP group. Various clinical outcomes, such as IKDC score, Lysholm score, Tegner score, knee anteroposterior and rotational laxity, range of motion and VAS, could not be improved with PRP application.

The PRP is associated with very limited role in improving knee outcomes following ACLR, and there is no indication for PRP procedures in ACLR at this stage.

## Graphical abstract

The graphical abstract including the main content of this review (left column), flowchart of study searching and selecting (top‐right grid), assessment of risk of bias (middle‐right grid), and barchart showing trials with significant /non‐significant findings (bottom‐right grid).

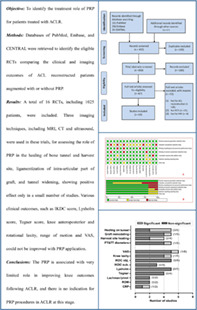

## Introduction

Anterior cruciate ligament (ACL) rupture is one of the most common injuries of knee joints, which often reports symptoms including pain, swelling, giving‐way, difficulty with athletic performance, and even accelerated degenerative changes on the knee joint.^1^, ^2^ ACL reconstruction (ACLR) with various grafts is generally successful and predictable, on restoring the knee function and stability.^3^ However, one of the challenges of ACLR is the slow integration in the bone tunnel and ligamentization of intra‐articular part of graft, which is one of the factors causing long rehabilitation period before returning to full physical activity.^4^, ^5^ Additionally, the slow healing process of defect at harvest site has been recognized as a cause of persistent anterior knee pain even after many years.^6^ Although the treatment role of platelets‐rich plasma (PRP) remains unclear, it has been provided with the aim of accelerating the maturation of the graft and healing processes of bone tunnel and donor site.^7^ Theoretically, after applying of PRP to the ACL‐reconstructed patients, a myriad of growth factors (GFs) and proteins would be released to the local environment, which could potentially accelerate the tissue regeneration. Platelets, as one of the best sources of growth factors, could release large amounts of activated microgranules rich in GFs during fibrin clot formation. Among these GFs, many have been proven to be involved in musculoskeletal tissue repair, including platelet‐derived growth factor (PDGF), transforming growth factor beta (TGF‐β), insulin growth factor (IGF), vascular endothelial growth factor (VEGF), and so on.^8^ These proteins regulate the processes of tissue healing, chemotaxis, proliferation, differentiation, angiogenesis and removal of tissue debris.^9^

There are two primary biological processes that take place after ACLR, including graft integrity in bone tunnel and ligamentization of the intra‐articular part of the graft.^10^, ^11^ The graft healing in the bone tunnels always starts with an acute inflammatory response when the tunnels are filled with blood from the drilled bone wall immediately after ACLR. This process mainly accompanied with edema, recruitment of neutrophils, macrophages and mesenchymal‐cell, as well as matrix synthesis, in the tendon bone interface. And then, in the chronic phase of the inflammatory response, the monocytes and stem cells initiate angiogenesis and regeneration of hypervascular granulation tissue interface between the graft and bone tunnel. The pattern of change taking place in the body of transplanted tendon is described as ligamentization, which mainly includes the stages of necrosis, swelling, revascularization, fibroblastic invasion and synthesis and maturation of collagen fibers with ligament reformation.^12^ Various GFs have participated in the entire process of ACL repairing following reconstruction, especially for PDGF and TGF. PRP has been recognized as a promising applicator of multiple GFs, and used for many indications in several fields of surgery, particularly for repairing of tissue damage and healing of skin and bone defects.^13^, ^14^, ^15^

Controversial results have been reported in the former literature referring to the potential treatment effect of PRP in ACLR.^16^, ^17^, ^18^, ^19^, ^20^, ^21^ Several culture studies have proven an increase on cellular component and collagen levels in tendon tissues with the use of PRP.^16^, ^17^ In canine model, Murray *et al*.^18^ found significant improvement on the biomechanical properties of the ACL after applying of collagen‐PRP hydrogel in particle ACL rupture. In the study of Xie *et al*.^19^ PRP was shown to be effective on promoting synthesis of extracellular matrix in dog models after ACLR. However, the treatment effect of PRP has mainly been evaluated on biomechanical and histological aspects using animal models. Other studies with a high level of evidence have not confirmed the role of PRP in patients treated with ACLR. Nin *et al*.^20^ evaluated the role of additional PDGF in primary ACLR with bone‐patellar tendon‐bone (BPTB) allograft, and found no discernable clinical or biomechanical effect at 2 years' follow‐up. In a previous systematic review by Hexter *et al*.^21^ clinical and preclinical studies evaluated biological augmentation of graft healing in ACLR were narratively synthesized, demonstrating mixed clinical outcomes according to the available suboptimal‐quality studies. Moreover, as a category of minimally manipulated tissue which is produced from autologous blood, the PRP used in clinical practice have a large inherent variability, due to the variation on the concentrations of platelets and growth factors in the peripheral blood, and the divergent preparation (e.g., the volume of blood, anticoagulant methods, processing systems, the speed and duration of spin cycles, format as liquid/gel, etc.) procedures.^22^ So, there remains an ambiguous understanding on the biological behavior of PRP in the procedure of ACLR.

In this study, we set out to perform a systematic review based on the evidence from randomized controlled trials (RCTs), with the aim of: (i) reviewing the application of PRP in the procedure of ACLR; (ii) assessing the clinical outcomes, including knee functional and stability evaluations, of ACLR with the application of PRP; and (iii) assessing the imaging outcomes of ACLR after PRP applying, such as healing of bone tunnels, graft remodeling, donor site healing and tunnel widening, basing on MRI, CT or ultrasound.

## Materials and Methods

This systematic review was carried out according to the guidelines outlined in the Preferred Reporting Items for Systematic Reviews and Meta‐analysis (PRISMA) statement, and the Minimum Information for Studies Evaluating Biologics in Orthopaedics (MIBO).^23^, ^24^ The PRISMA checklist and the MIBO checklist for clinical studies evaluating PRP are available in Supporting information Appendix S1 and S2.

### Study Eligibility and Selection

Studies would be included for eligibility according to the following criteria: (i) P (participants)—patients diagnosed with symptomatic unstable knee due to ACL rupture; (ii) I (intervention) —ACLR with various tendon grafts plus biological augmentation with PRP; (3) C (comparison) —exclusively ACLR without PRP application or any other bioaugmentation (such as stem cells/ amnion/ hyaluronic acid); (iv) O (outcomes) —image evaluations (by MRI, CT or ultrasound) or clinical evaluations on the reconstructed knee; and (v) S (study) —rigorously designed RCTs. Studies designed as observational or non‐randomized clinical studies, reviews, experimental studies, case reports, case series and letters to editors would be excluded. There is no limitation on the PRP injection sites, including femoral/ tibial tunnels, inside the graft, suprapatellar joint, donor site, or intra‐articular injection. The publication language was restricted to English.

Three databases, including PubMed, Embase and the Cochrane Central Register of Controlled Trials (CENTRAL), were systematically retrieved by two independent reviewers, from the inception to October 2021. The detailed searching strategies are presented in Supporting information Appendix S3. Other potential articles were hand searched after screening the references lists of the included studies. The initially retrieved studies were put together for duplicate checking. After excluding the duplicated studies, the titles/abstracts and full‐texts of the remained records were screened successively for final eligibility, by two individual reviewers.

### Data Extraction and Risk of Bias Assessment

Data was extracted by two reviewers independently, and entered into a pre‐built Microsoft Excel spreadsheet, including the following items: (i) study details—lead author's name, publication year, study period, follow‐up information and funding source; (ii) participants details—number of patients, number of dropped patients, percentage of male, and mean age; (iii) intervention information—application of PRP, preparation protocol of PRP, site, time point and volume of PRP application, graft type for ACLR, and fixation methods both in femoral and tibial sides; and (iv) outcomes information—image evaluations on the healing of bone tunnels, graft remodeling, donor site healing and tunnel widening, and clinical evaluations on knee stability and function, pain symptom by visual analogue scale (VAS), inflammatory parameters and so on. Cross‐checking on the collected data by the two reviewers was performed to detect potential disagreements, which would be resolved by a third senior reviewer. All of the recorded data were displayed in tables or narratively synthesized.

The risk of bias assessment of the included studies were conducted by two researchers independently using the Cochrane Collaboration tool.^25^ This tool is specially designed for assessing the risk of the following biases for RCTs: randomization sequence generation, allocation concealment, blinding of both participants and personnel, blinding of outcome assessment, incomplete outcome data, selective reporting and other bias. Each item is set as unclear, low risk of bias or high risk of bias.

## Results

### Study Selection

**Fig. 1:**
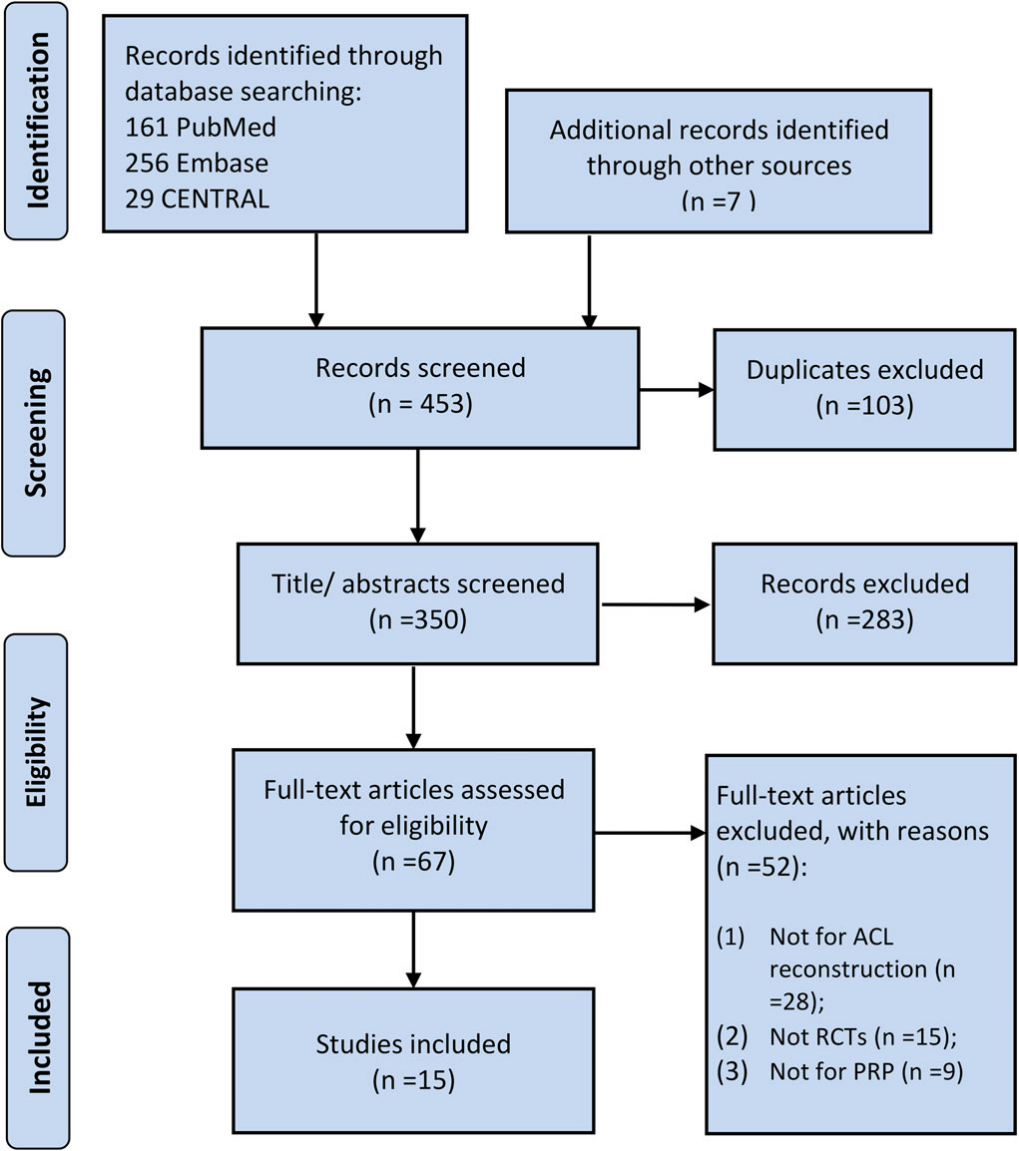
PRISMA flowchart for the searching and selecting of studies.

Figure 1 shows the flow chart of study searching and selecting. The initial retrieval in the databases yielded a total of 446 potential records, and seven additional records were identified through manual search. A total of 103 duplicates were excluded, leaving 350 titles/abstracts for further screening. Then, only 68 records remained for full‐text assessing, among which 52 articles were not relevant to the inclusion criteria. Finally, a total of 16 RCTs^20^, ^26^, ^27^, ^28^, ^29^, ^30^, ^31^, ^32^, ^33^, ^34^, ^35^, ^36^, ^37^, ^38^, ^39^, ^40^ were eligible for our systematic review.

### Characteristics of the Included Studies

Summary of the characteristics of eligible studies is displayed in Table 1. A total of 1025 patients, with a mean age between 22.7–37.2 years were involved in the primary trials. Of these, 577 patients (56.3%) were assigned in the treatment group with PRP, while 448 (43.7%) patients in the control group. Fourteen studies reported the percentage of male patients, including a total of 633 (75.2%) male and 209 (24.8%) female. In total, 85 patients were lost to the final follow‐up. Two‐arm studies predominated among the primary trials, while there were only two four‐arm^26^, ^30^ and one three‐arm^28^ study. In 11 of the trials,^20^, ^26^, ^28^, ^29^, ^30^, ^31^, ^34^, ^35^, ^36^, ^39^, ^40^ the PRP products were applied to the femoral tunnel, tibial tunnel, and inside the graft alone or with different combinations, at the end of the operation or intra‐operation. In the trial of Silva *et al*.^26^ they injected PRP in the femoral tunnel and intra‐articular at the end of surgery as well as 2 and 4 weeks post‐operatively, for one of the treatment group. In four of the studies,^27^, ^32^, ^33^, ^37^ PRP was applied to the harvest site of BPTB autograft intraoperative or at the end of operation. Seijas *et al*.^38^ percutaneously injected PRP into the suprapatellar joint for their patients. Different volumes of PRP were used in each site as reported in 12 studies.^26^, ^29^, ^30^, ^32^, ^33^, ^34^, ^35^, ^36^, ^37^, ^38^, ^39^, ^40^

**TABLE 1:** Summary of the characteristics of the included studies

Author/year	Study period	Treatment group	No. of pts.	Drop out	Male%	Mean age (years)	Injection site (s) and time points	Injection volume	Follow‐up periods	Graft type	Fixation methods (F = Femoral side, T = Tibial side)	Use of knee brace
Silva, 2009^26^	Nov. 2006 ‐Mar. 2008	Control	10	0	95.0 (overall)	26.8 ± 5.3 (overall)	—	—	Mean: 97.2 ± 6.8 d (84–117 d)	Double‐bundle HT autograft	F: EndoButton CL; T: bioabsorbable interference screw	1 week post‐op
		PRP	10	0			FT (end of surgery)	1.5 ML in each FT at the end of surgery				
		PRP	10	0			FT & IA (end of surgery, 2 & 4 w post‐op)					
		PRP gel	10	0			FT (end of surgery)					
Cervellin, 2012^27^	2008–2009	Control	20	0	NA	22.7 ± 3.5	—	—	Time‐points:12 m	BPTB autograft	NA	No use
		PRP gel	20	0		22.9 ± 4.3	Donor site (end of surgery)	NA				
Valentí Azcárate, 2014^28^	NA	Control	50	0	76.0	26.1 (15–59)	—	—	Time‐points: 1 d, 10 d, 3 m, 6 m, 12 m	PT allograft	F: biodegradable cross pins; T: biodegradable interference screw	10 days post‐op
		PRP	50	0	80.0	26.1 (14–57)	TT & inside the graft (intra‐op)	NA				
		PRGF	50	0	86.0	27.4 (16–50)						
Vogrin, 2010^29^	Feb. 2008 ‐Oct. 2008	Control	25	5	64.0	32.6 ± 12.3	—	—	Time‐points: 4–6 w, 10–12 w	Double‐looped HT autograft	F:2 bioabsorbable cross pins; T:1 bioabsorbable interference screw	No use
		PRP gel	25	4	60.0	37.2 ± 8.4	FT, TT & inside the graft (intra‐op)	4 ml: graft; 1 ml: FT; 1 ml: TT				
Orrego, 2008^30^	Jan. 2005 ‐Dec. 2006	Control	27^a^	8 in total	85.0 (overall)	30.0 (15–57) (overall)	—	—	Time‐points: 3 m, 6 m	4‐strand HT autograft	F: biodegradable transfixing pin; T: biodegradable interference screw	No use
		PRP	26^a^				FT & inside the graft (intra‐op)	5 ml: between strands; 1 ml: FT				
		Bone plug	28^a^									
		PRP + Bone plug	27^a^									
Rupreht, 2013^31^	NA	Control	25	5	64.0	32.6 ± 12.3	—	—	Time‐points: 1 m, 2 m, 5 m, 6 m	Double‐looped HT autograft	F: 2 bioabsorbable cross pins; T: 1 bioabsorbable interference screw	NA
		PRP gel	25	4	60.0	37.2 ± 8.4	FT, TT & inside the graft (intra‐op)	NA				
Nin, 2009^20^	NA	Control	50	0	76.0	26.6 (15–59)	—	—	Time‐points: 3 m, 6 m, 12 m & yearly thereafter	PT allograft	F: 2 bioabsorbable cross pins; T: 1 bioabsorbable interference screw	10 days post‐op
		PRP gel	50	0	80.0	26.1 (14–57)	TT & inside the graft (intra‐op)	NA				
Seijas, 2013^38^	During 2009	Control	20	0	NA	NA	—	—	Time‐points: 1 m, 2 m, 4 m, 6 m, 9 m, 12 m, 24 m	BPTB autograft	NA	NA
		PRGF	23	1			Donor site (end of surgery)	4 ml				
de Almeida, 2012^33^	Nov. 2008 ‐Feb. 2010	Control	15	3	93.0	23.1 (15–34)	—	—	Time‐point: 6 m	BPTB autograft	F: absorbable transverse pin; T: absorbable interference screw	No use
		PRP gel	12	2	83.0	25.8 (18–44)	Donor site (intra‐op)	20 ~ 40 ml				
Vadalà, 2013^34^	NA	Control	20	0	100	34.5 (18–48) (overall)	—	—	Median:14.7 m (10–16 m)	4‐strand HT autograft	F: the Swing‐Bridge device; T: the Evolgate	No use
		PRP gel	20	0	100		FT, TT & inside the graft (intra‐op)	10 ml: FT & graft; 5 ml: TT				
Vogrin, 2010^35^	Feb. 2008 ‐Jun. 2008	Control	25	2	73.9	33.0 ± 12.5	—	—	Time‐points: 3 m, 6 m	Double‐looped HT autograft	F: 2 bioabsorbable cross pins; T: 1 bioabsorbable interference screw	No use
		PRP gel	25	3	59.1	35.4 ± 10.0	FT, TT & inside the graft (intra‐op)	6 ml				
Mirzatolooei, 2013^36^	Feb. 2011 ‐Feb. 2012	Control	25	2	96.0	26.9 (18–40)	—	—	Time‐points: 3 m	4‐strand HT autograft	F: cross‐pin; T: bioabsorbable interference screw	2 weeks post‐op
		PRP	25	2	87.0	26.4 (18–40)	FT & TT (intra‐op)	2 ml: FT; 1.5 ml: TT				
Walters, 2018^37^	2011–2015	cancellous bone chips	29	14	52.0	30.0 ± 12.0 (overall)	Donor site (intra‐op)	—	Time‐points: 3 m, 6 m, 12 m, 24 m	BPTB autograft	F&T: titanium cannulated interference screws	NA
		PRP soaked cancellous bone chips	30	10	37.0		Donor site (intra‐op)	3‐5 ml: mixed with cancellous bone chips				
Seijas, 2013^38^	Jan. 2009 ‐Jul. 2009	Control	50	1	NA	NA	—	—	Time‐points: 4 m, 6 m, 12 m	BPTB autograft	F&T: hydroxylapatite screws	Immobilization for 1 week with 2 plaster splints
		PRP	50	1			Suprapatellar joint (end of surgery)	8 ml				
Rupreht, 2013^39^	NA	Control	25	5	26.0	32.6 ± 12.3	—	—	Time‐points: 1 m, 2.5 m, 6 m	Double‐looped HT autograft	F: 2 bioabsorbable cross pins; T: 1 bioabsorbable interference screw	NA
		PRP gel	25	4	60.0	37.2 ± 8.4	FT, TT & inside the graft (intra‐op)	1 ml: FT; 1 ml: TT; 3 ml: graft				
Starantzis, 2014^40^	Dec. 2007 ‐Jun. 2010	Control	30	4	74.5 (overall)	31.3 ± 8.0	—	—	Time‐points: 1 m, 12 m	4‐strand HT autograft	F: Crosspin /Endobutton; T: a biodegradable interference screw + bone bridge suture anchoring	NA
		PRP	30	5		29.4 ± 7.3	FT & inside the graft (intra‐op)	3 ml: FT; 3 ml: graft				

Abbreviations: BPTB, bone‐patellar tendon‐bone; FT, femoral tunnels; HT, hamstring tendon; NA, not available; PRGF, plasma rich in growth factors; PRP, platelet‐rich plasma; PT, patellar tendon; TT, tibial tunnel.

^a^ Only the number of patients at the final follow‐up was reported in the primary studies.

^a^ *Note:* Only the number of patients at the final follow‐up was reported in the primary studies. Letters “d, w, and m” accompanying time points/follow‐up periods represent days, week and month respectively.

Regarding the graft types selected for ACLR, four‐strand HT autograft was used in eight studies,^29^, ^30^, ^31^, ^34^, ^35^, ^36^, ^39^, ^40^ while BPTB autograft, double‐bundle HT autograft and PT allograft were used in five,^27^, ^32^, ^33^, ^37^, ^38^ one^26^ and two^20^, ^28^ studies respectively. The internal fixation methods in the femoral and tibial tunnels were reported in 14 studies.^20^, ^26^, ^28^, ^29^, ^30^, ^31^, ^33^, ^34^, ^35^, ^36^, ^37^, ^38^, ^39^, ^40^ The cross‐pin, EndoButton device, and interference screw were used for femoral tunnel fixation in 10,^20^, ^28^, ^29^, ^30^, ^31^, ^33^, ^35^, ^36^, ^39^, ^40^ two^26^, ^34^ and two^37^, ^38^ studies respectively, while interference screw was used for all of the tibial tunnel fixation. Detailed rehabilitation protocols were available in 11 studies.^20^, ^26^, ^27^, ^28^, ^29^, ^30^, ^33^, ^34^, ^35^, ^36^, ^38^ Knee bracing (or plaster splint in one study^38^) was applied for early immobilization in four studies,^20^, ^26^, ^28^, ^36^ and accelerated rehabilitation protocols were applied without using of knee braces in the other six studies.^27^, ^29^, ^30^, ^33^, ^34^, ^35^ The summary of funding sources of each trial is available in Supporting information Appendix S4. The funding information was available in seven studies,^27^, ^30^, ^33^, ^35^, ^36^, ^37^, ^40^ five^27^, ^30^, ^33^, ^37^, ^40^ of which were partially supported by some funders.

Table 2 represents the preparation protocols for PRP in the included studies. The median volume of whole blood drawn from patients was 40 (range: 10–450 mL). In 11 of the studies,^20^, ^26^, ^27^, ^28^, ^29^, ^30^, ^32^, ^33^, ^35^, ^37^, ^40^ anticoagulant process was reported, which was predominately conducted with citric acid or citrates. Various processing devices were selected for PRP preparation, and double‐spinning process was used in the studies of Nin *et al*.^20^ and Valentí Azcárate *et al*.^28^ Platelet counts in whole blood and/ or PRP were performed for post‐preparation analysis in six studies.^20^, ^27^, ^29^, ^31^, ^33^, ^35^ PRP in liquid and gel‐like PRP (activated with thrombin or CaCl_2_ solution) were applied in two studies^26^, ^36^ and 14 studies,^20^, ^26^, ^27^, ^28^, ^29^, ^30^, ^31^, ^32^, ^33^, ^34^, ^35^, ^36^, ^39^, ^40^ respectively.

**TABLE 2:** Harvest procedures, spin parameters and characteristics of PRP in the included studies

Author/year	WB Volume	Anticoagulant	Processing machine	Spin		Post‐preparation analysis	PRP activation	PRP format
				Speed (r/min)	Time (min)			
Silva, 2009^26^	27 ml	citric acid (3 ml)	the Mini GPS III Kit (Biomet®, Warsaw, IN, USA)	3200	15	no	thrombin (6 ml from 12 ml citrated WB, in group D)^**a**^	liquid/ gel^a^
Cervellin, 2012^27^	54 ml	citric acid (ACD‐A, 6 ml)	the Gravitational Platelet Separation II (GPS®) system (Biomet Biologics, Inc, Warsaw, IN, USA)	3200	15	platelet count in WB	thrombin (2.5 ml from 10 ml citrated WB) + CaCl_2_ (0.5 ml)	gel
Azcárefate, 2014^28^	40 ml	sodium citric (3.8%, wt/vol)	(1) Beckman J‐6B, Beckman Coulter Spain; (2) PRGF‐Endoret® Technology (BTI System II)	(1) 3000 + 1000; (2) 1800^**b**^	(1) 8 + 6; (2) 8^**b**^	no	CaCl_2_ (10%, 0.05 ml per 1 ml PRP)	gel
Vogrin, 2010^29^	52 ml	calcium citric (10%, 8 ml)	the Magellan (Medtronic Biologic Therapeutics and Diagnostics, Minneapolis, MN, USA) autologous platelet separator	NA	NA	Platelet count in PRP	thrombin	gel
Orrego, 2008^30^	57 ml	unknown anticlotting agent (3 ml)	the Biomet GPS II kit (Biomet®, Waraw, IN, USA)	NA	15	no	thrombin (collected from 10 ml WB) + CaCl_2_ (10:1, vol/vol)	gel
Rupreht, 2013^c^, ^31^	NA	NA	NA	NA	NA	platelet counts in WB and PRP	NA	gel
Nin, 2009^20^	40 ml	citric acid	Beckman J‐6B, Beckman Coulter Spain, Madrid, Spain	3000 + 1000^d^	8 + 6^d^	platelet counts in WB and PRP	CaCl_2_ (10%, 0.05 ml per 1 ml PRP)	gel
Seijas, 2013^38^	10 ~ 20 ml	trisodium citrate (10%)	NA	160G^**e**^	6	no	CaCl_2_ (10%, 0.05 ml per 1.2 ml PRP)	gel
de Almeida, 2012^33^	450 ml^**f**^	citrate (10%)	Haemonetics MCS + 9000 cell separator with a specific kit for platelet apheresis 995‐E (Haemonetics Corp, Braintree, MA, USA)	NA	NA	platelet and WBC counts in WB and PRP	thrombin+CaCl_2_ (0.8 ml)	gel
Vadalà, 2013^34^	10 ml	NA	the PRP Fast Biotech kit (MyCells® PPT‐Platelet Preparation Tube)	NA	NA	no	thrombin+Ca‐gluconate (10%)	gel
Vogrin, 2010^35^	52 ml	calcium citrate (8 ml, 10%)	the Magellan autologous platelet separator (Medtronic Biologic Therapeutics and Diagnostics, Minneapolis, MN, USA)	NA	NA	platelet counts in WB and PRP	thrombin	gel
Mirzatolooei, 2013^36^	10 ml	NA	a double syringe system (Arthrex)	1500	5	no	no	liquid
Walters, 2018^37^	10 ml	citric acid (ACD‐A, 1 ml)	a PRP separation kit and centrifuge system (ACP PRP; Arthrex)	1500	5	no	CaCl_2_ (0.25 ml)	gel
Seijas, 2013^38^	NA	NA	a PRGF technique (BTI Systems Vitoria, Spain)	NA	NA	no	NA	NA
Rupreht, 2013^39^	NA	NA	NA	NA	NA	no	thrombin	gel
Starantzis, 2014^40^	65 ml	citric acid (ACD‐A, 5 ml)	the Biomet GPS III kit (Biomet, Warsaw, IN, USA)	3200	15	no	thrombin (collected from 10 ml WB) + CaCl_2_ (10:1, vol/vol)	gel

Abbreviations: NA, not available; PRP, Platelet‐Rich Plasma; WB, whole blood; WBC, white blood cell.

^a^ Note: the Clotalyst™ autologous thrombin collection system (Biomet®) was used to obtain thrombin for clotting of platelets only for patients in group D, while liquid PRP was applied in groups B and C.^20^

^b^ double‐spin procedure and single‐spin procedure were performed in the two groups respectively.

^**c**^ PRP preparation was similar to that previously described in Nin *et al*.^20^ and Orrego *et al*.^30^. The detailed preparation process was not available.

^d^ double‐spinning process was used to collect the platelet‐enriched gel.

^**e**^ relative centrifugal field (RCF, recorded in G) was used to present the speed of spin.

^**f**^ the red blood cells and PPP (up to 400 mL) were returned to the patient from their collection bags through the peripheral venous access.

**Fig. 2:**
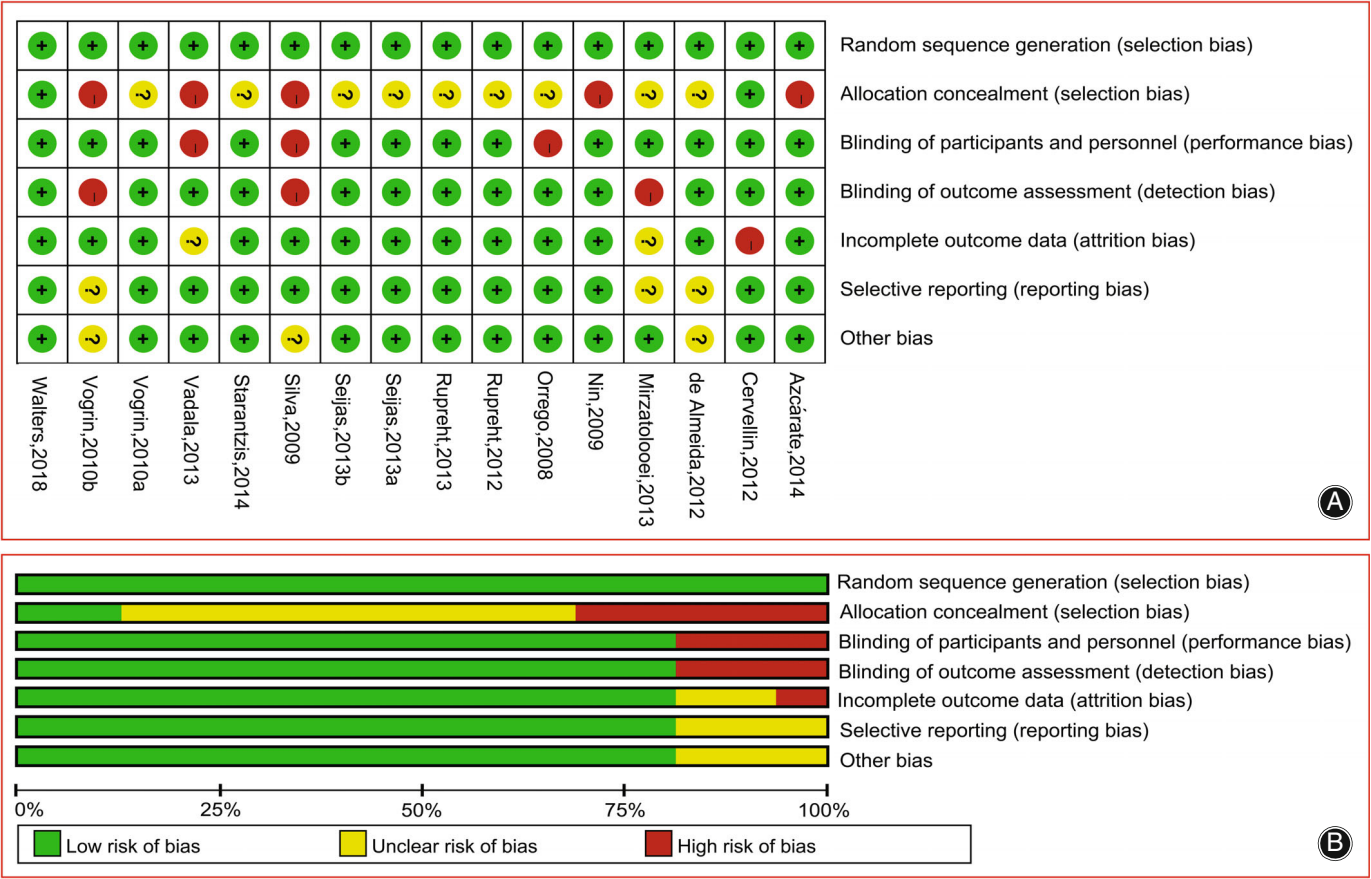
The risk of bias graph for each study and the summaries of the risk of bias. Generally, the included studies were of low risk of bias, but the information about allocation concealment was unclear in many included studies.

Figure 2 represents the risk of bias graph for each study and the summaries of the risk of bias. Generally, the included studies were of low risk of bias, but the information about allocation concealment was unclear in many included studies.

### Qualitative Synthesis of the Outcome Evaluations

**Fig. 3:**
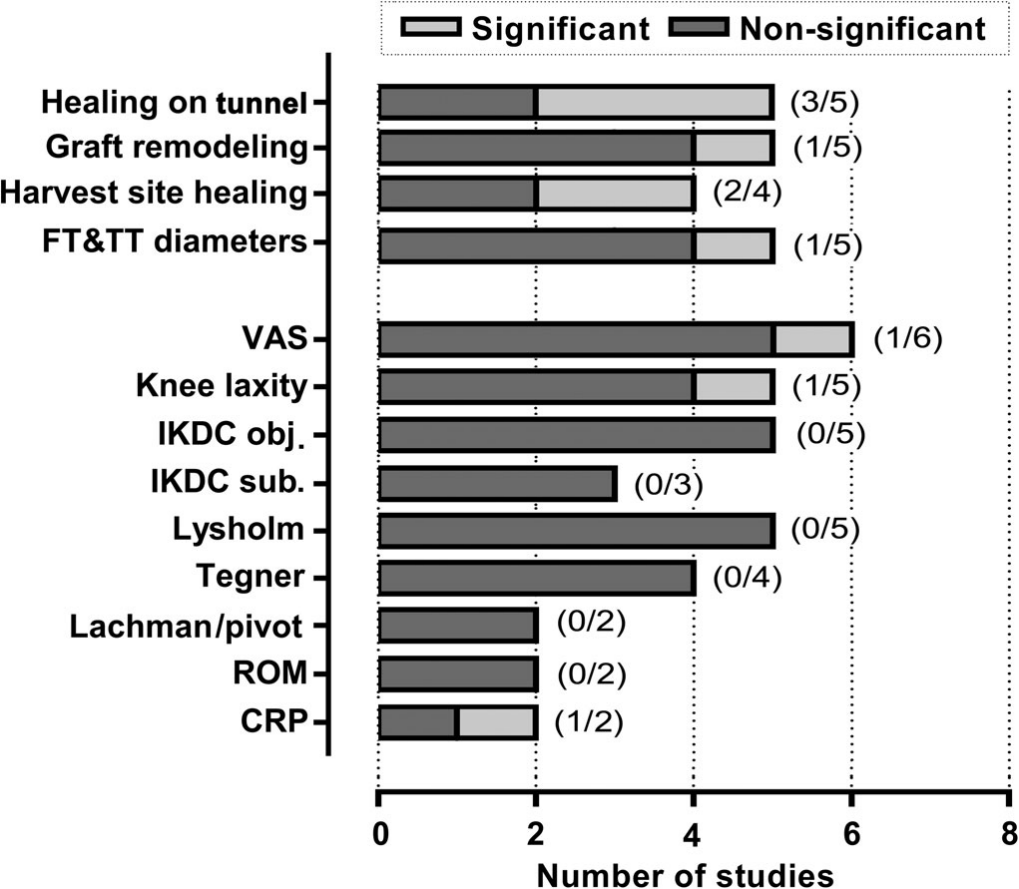
Number of trials with significant findings for various imaging and clinical assessments. In the image evaluations, significant improvement on graft remodeling, bone tunnel healing, harvest site healing and bone tunnel diameters were reported in one of five (20.0%), three of five (60.0%), two of four (50.0%) and one of five (20.0%) studies respectively, for PRP group. Concerning the clinical evaluations, one of six studies (16.7%) showed significantly lower VAS in the treatment group with PRP than control group. The knee anteroposterior stability was shown to be increased in one of five studies (20.0%) following additional applying of PRP. No significance was found for the knee function (IKDC scores, Lysholm score, Tegner score, and ROM) and rotational stability (Lachman and pivot‐shift tests) evaluations at various points of follow‐up. Concerning the post‐operative inflammatory parameters, CRP was found to be significantly decreased in one of two studies (50.0%) at one day post‐operation.

Table 3 shows the main outcomes and the significant findings in each primary study. Image assessment on the treatment effect of PRP was performed in 15 RCTs,^20^, ^26^, ^27^, ^28^, ^29^, ^30^, ^31^, ^32^, ^33^, ^34^, ^36^, ^37^, ^38^, ^39^, ^40^ with MRI in 12 studies,^20^, ^26^, ^27^, ^28^, ^29^, ^30^, ^31^, ^33^, ^37^, ^38^, ^39^, ^40^ CT in two studies,^34^, ^36^ and ultrasound in one study.^32^ Table 4 presents the types of MRI used for outcome assessment. Several types of MRI imaging techniques were performed, mainly including the proton density‐weighted images, T1/T2‐weighted images, contrast‐enhanced images with intravenous administration of gadolinium or paramagnetic contrast medium Gd‐DTPA, and sometimes combining with spectral fat saturation. In general, MRI was mainly used for evaluating the signals of the bone tunnels, the intra‐articular part of the graft and the defect on BPTB harvest site, for assessing the processes of bone tunnel healing, graft maturation and donor site healing. Additionally, it was also used to assess the widening and direction of femoral and tibial tunnel, and tibial anterior translation. CT was only used for measuring the diameters of femoral and tibial tunnels. The ultrasonography testing on the vascularization of PT and state of defect repair at the harvest site was used for assessing harvest site healing. In these image evaluations, significant improvement on graft remodeling, bone tunnel healing, harvest site healing and bone tunnel diameters were reported in one of five (20.0%), three of five (60.0%), two of four (50.0%) and one of five (20.0%) studies respectively, for PRP group. Figure 3 shows the number of studies reported significant and non‐significant results for the image and clinical evaluations.

**TABLE 3:** Summary of main results of included studies

Author/year	Outcome measures	Significant findings
Silva, 2009^26^	Image assessment: MRI signal of the FIZ	None
Cervellin, 2012^27^	Image assessment: MRI evaluations on harvest site healing; Clinical assessment: anterior knee pain and kneeling pain by VAS, and VISA scale	VISA scores were significantly higher in the patients treated with PRP
Valentí Azcárate, 2014^28^	Image assessment: MRI evaluations on intensity, thickness, and uniformity of graft, direction of TT and FT, and tibial anterior translation; Clinical assessment: VAS, side‐to‐side difference by KT‐1000, IKDC objective score, CRP and PER	Significant improvements in swelling and inflammatory parameters were found for PRGF group at 1d post‐op
Vogrin, 2010^29^	(1) Image assessment: MRI evaluations on revascularization rate in FIZ & intra‐articular part of graft, and diameters of FT & TT	(1) Significantly higher level of vascularization in FIZ was shown in PRP group, at 4–6 weeks
Orrego, 2008^30^	Image assessment: MRI evaluations on graft signal intensity in FT, presence of an interface between graft and FT and tunnel widening; Clinical assessment: Lysholm score, IKDC objective score	Increased number of patients presented low‐intensity signal in PRP group than control group at 6 months; Tunnel widening was decreased in bone plug group than control group at 6 months
Rupreht, 2013^31^	Image assessment: apparent diffusion coefficient (ADC) values, contrast enhancement gradient (Genh), enhancement factor (Fenh) values by diffusion weighted imaging (DWI) and with dynamic contrast‐enhanced imaging (DCE‐RI) in TT	ADC value in the PRPG group was significantly lower than in the control group at 1 month; Genh was significantly higher in the PRPG group at 2.5 and 6 months
Nin, 2009^20^	Image assessment: MRI evaluations on graft intensity, thickness and uniformity, the direction of TT & FT, tibial anterior translation, and position of PCL; Clinical assessment: VAS, knee laxity by KT‐1000, IKDC objective score and CRP	None
Seijas, 2013^38^	Image assessment: Ultrasound evaluations on vascularization of the tendon and the state of repair at the harvest site	Significantly higher scores of maturity were found in PRGF group than control group, at 4m post‐op
de Almeida, 2012^33^	Image assessment: MRI evaluations on harvest site healing; Clinical assessment: VAS, Lysholm score, IKDC subjective score, Kujala score, Tegner score and isokinetic strength measurements of quadriceps and hamstring muscles	PT gap area at harvest site was significantly smaller, and VAS was lower in PRP group, at 6m post‐op
Vadalà, 2013^34^	Image assessment: CT evaluations on diameters of FT & TT; Clinical assessment: ROM, Lachman and pivot‐shift tests, Lysholm score, Tegner score, IKDC objective score, and knee laxity by KT‐1000	None
Vogrin, 2010^35^	Clinical assessment: Tegner score, Lysholm score, IKDC score and knee laxity by KT‐2000 arthrometer	Improvement on knee anteroposterior stability at 6 month post‐op was significantly higher in PRP group
Mirzatolooei, 2013^36^	Image assessment: CT evaluations on diameter at the aperture and in the middle of tunnels; Clinical assessment: ROM, knee laxity by KT‐1000, and VAS	None
Walters, 2018^37^	Image assessment: MRI evaluations on graft site defect and anteroposterior dimensions of patellar tendon; Clinical assessment: VAS and IKDC subjective score	None
Seijas, 2013^38^	Image assessment: MRI evaluations on stages of the grafts remodeling	None
Rupreht, 2013^39^	Image assessment: MRI evaluations on percentage of TT wall cortical bone	Significant increase on average percentage of TT wall cortical bone for PRP group than control group, at 2.5m and 6m post‐op
Starantzis, 2014^40^	Image assessment: MRI evaluations on FT diameters; Clinical assessment: Lysholm score, Tegner score, Rolimeter assessment and pivot‐shift test	Significant decrease on the tunnel dilation at the middistance of the FT in PRP group, at 12m post‐op

Abbreviations: CRP, C‐response protein; FIZ, fibrous interzone; FT, femoral tunnels; PER, knee perimeters; PRGF, plasma rich in growth factors; TT, tibial tunnel; PRP, platelet‐rich plasma; ROM, range of motion.

**TABLE 4:** Types of MRI used for outcome assessment

Author/year	Types of MRI
Silva, 2009^26^	Proton density weighted image with spectral fat saturation; T_1_ weighted image with spectral fat saturation after administration of intravenous gadolinium
Cervellin, 2012^27^	T_1_ and T_2_‐weighted images
Valentí Azcárate, 2014^28^	Orthogonal proton density‐weighted images (axial, sagittal, and coronal); T_1_ and T_2_‐weighted images
Vogrin, 2010^35^	Contrast‐enhanced T_1_‐weighted images after intravenous administration of paramagnetic contrast medium Gd‐DTPA
Orrego, 2008^30^	T_2_‐weighted images (sagittal and axial)
Rupreht, 2013^31^	Proton density weighted images; Dynamic contrast‐enhanced (DCE‐MRI) images after intravenous administration of paramagnetic contrast medium Gd‐DTPA
Nin, 2009^20^	Orthogonal proton density‐weighted images (axial, sagittal, and coronal); T_1_ and T_2_‐weighted images
de Almeida, 2012^33^	T_2_‐weighted fat‐saturated fast spin‐echo images (axial); T_2_‐weighted fat‐saturated images (sagittal); Intermediate‐weighted fast spin‐echo images
Walters, 2018^37^	Fluid‐sensitive images (axial)
Seijas, 2013^38^	NA
Rupreht, 2013^39^	Proton‐density weighted fat‐suppressed images
Starantzis, 2014^40^	T_1_‐weighted images (coronal and axial); Proton density weighted (sagittal) /T_2_‐weighted images; STIR (coronal) or proton density‐weighted (coronal) images with spectral fat saturation; T_1_‐weighted images with spectral fat saturation after administration of intravenous gadolinium

Clinical outcomes assessments were available in 10 studies, mainly including VAS, knee anteroposterior laxity by KT‐1000/KT‐2000, IKDC subjective and objective score, Lysholm score, Tegner score, Lachman test, pivot‐shift test, range of motion (ROM) and concentration of inflammatory parameters such as C‐reactive protein (CRP). Among these studies, one of six studies (16.7%) showed significantly lower VAS in the treatment group with PRP than control group. The knee anteroposterior stability was shown to be increased in one of five studies (20.0%) following additional applying of PRP. No significance was found for the knee function (IKDC scores, Lysholm score, Tegner score, and ROM) and rotational stability (Lachman and pivot‐shift tests) evaluations at various points of follow‐up. Concerning the post‐operative inflammatory parameters, CRP was found to be significantly decreased in one of two studies (50.0%) at one day post‐operation.

## Discussion

The main finding was that only a few publications demonstrated a positive effect of PRP on accelerating the maturation process of tendon graft and healing processes on bone tunnel and the harvest site of autologous graft, and the clinical outcomes could hardly be significantly improved following application of PRP.

### Application and Effectiveness of PRP for Tendon Healing

It has been reported that the long period of healing in the interzone between bone tunnel and graft makes up one of the factors to delay the return to pre‐injury activity, and there is also an existent relationship between harvest site healing time and anterior knee pain.^4^, ^5^, ^6^ Though the treatment role of PRP in ACL‐reconstructed patients has not been definitely identified, it has been applied with the anticipation of accelerating the recovering processes. In many former cytological, histological or biomechanical studies, PRP was shown to have positive effects on promoting tendon healing at the injured site by promote cell proliferation and tissue regeneration.^16^, ^41^, ^42^, ^43^, ^44^, ^45^ Chan *et al*.^41^ studied the effects of basic fibro‐blast growth factor (bFGF) on cell proliferation, type III collagen expression, ultimate stress and the pyridinoline content in the early stages of healing in rat patellar tendons, and found a dose‐dependent increase in the number of proliferating cells and the level of expression of type III collagen at 7 days post‐injury. Anaguchi *et al*.^42^ have also shown that the tangent modulus and the tensile strength of regenerated tissue in the patellar tendon after resecting the central portion could be significantly improved by TGF‐β injections. In a cell culture study of de Mos *et al*.^16^ the PRP was reported to stimulate cell proliferation and total collagen production, and slightly increase the expression of matrix‐degrading enzymes and endogenous growth factors. These pre‐clinical studies were generally associated with tendon‐to‐tendon healing at the site of injury, that is known as the ligamentization process. However, the ACLR was mainly about the tendon‐to‐bone (without bone block) or bone‐to‐bone (with bone block) healing. Xie *et al*.^43^ indicated that PRP application could promote the revascularization and reinnervation after ACLR in a dog model, which might explain the enhancing effect of PRP on ACL graft maturation. In the study of Zhang *et al*.^44^ autologous PRP combined with gelatin sponge was demonstrated to be effective in improving the tendon‐to‐bone interface healing and structure formation after ACLR with semitendinosus autograft in rabbit model.

### Clinical and Imaging Outcomes Following PRP Application

In human studies, the histologic and biomechanical data could not be obtained due to ethical implications. Thus, MRI and other imaging techniques, such as CT, have been used for assessing the treatment result of PRP, including graft revascularization, healing of the fibrous interzone between bone tunnel and graft, bone tunnel widening, and healing of the donor site. In this systematic review, based upon the available high‐level evidence from RCTs, less significant findings were demonstrated in the imaging evaluations following ACLR, which was very different to that was represented in histological and biomechanical studies.^26^, ^27^, ^36^, ^37^ In order to evaluate the role of PRP in the tendon‐to‐bone healing of the ACL reconstructed with HT, Silva *et al*.^26^ examined the MRI signal intensity of the fibrous interzone in the femoral tunnels for patients with or without applying PRP, and no difference was found between groups at 3 months after surgery. In the RCT of Mirzatolooei *et al*.^36^ they assessed the impact of PRP on the prevention of tunnel widening in ACLR using quadrupled autologous HT. No significant difference was found between the groups treated with or without PRP at 3 months post‐operatively, both for the femoral and tibial tunnels. Cervellin *et al*.^27^ also evaluated the effect of PRP on reducing subjective pain (VAS scoring) and accelerating the healing of bone and tendon defect at donor site (MRI analyses) after BPTB harvesting for ACLR, showing no effect of PRP in reducing the VAS pain score and accelerating healing of bone and tendon defect at 12‐month follow‐up. Similarly, Walters *et al*.^37^ also found similar levels of kneeling pain and patellar defect sizes at different follow‐up periods after ACLR with BPTB autograft, whether patients were randomized to receive PRP in their patellar defect or not. Additionally, almost all of the trials assessing the clinical outcomes failed to find a positive treatment effect of PRP for ACL‐reconstructed patients.^20^, ^26^, ^34^, ^36^, ^37^, ^40^ Vadalà *et al*.^34^ evaluated the efficacy of PRP in reducing femoral and tibial tunnel enlargement, and improving knee outcomes including Tegner score, Lysholm score, IKDC objective score, as well as knee laxity by KT‐1000 arthrometer, in patients operated on for ACLR with HT, and no difference was found between the treatment groups. It could be speculated that though *in vitro* and animal studies have widely verified the positive role of PRP in tendon regeneration and healing, the ACLR in human patients is generally complex and the treatment outcomes could be affected by multiple factors, such as graft types, fixation methods, postoperative rehabilitation protocols, and so on. Large variations exist during the procedures of PRP preparation with various commercially available preparation systems. Thus, it is not applicable to include various PRP preparations in one general concept. Moreover, clinical evaluations carried out were mainly limited to short‐term follow‐up (as shown in Table 1, all of the studies followed for less than 24 months with seven studies^26^, ^30^, ^31^, ^33^, ^35^, ^36^, ^39^ no more than 6 months), which probably prevents an accurate assessment on long‐term benefit of PRP.

### Limitations

This systematic review, although based on RCTs with high level of evidence, has some potential weaknesses. First, it is not feasible to perform quantitative syntheses for the treatment outcomes, due to the existence of significant clinical heterogeneity among the primary trials, which includes different PRP patterns, volumes and application sites, different tendon graft types, fixation methods, rehabilitation protocols and follow‐up, and so on. However, it is necessary to obtain an exact quantitative pooling result about the effectiveness of PRP in ACLR. Hence, to decrease the diversities of studies and increase the possibility of synthetic analysis, a standard guideline about PRP application during ACLR is required. Additionally, future trials should strictly follow the MIBO checklist for clinical studies evaluating PRP when reporting the research. Then, only imaging and clinical assessments were available for evaluating the treatment effect of PRP for ethical consideration, while histological and biomechanical data could not be obtained for patients operated with ACLR.

## Conclusion

In summary, although some trials have identified a positive effect of PRP on imaging outcomes, this systematic review failed to demonstrate a discernable treatment effect of PRP according to clinical assessments. Thus, there is at this stage, no indication for the benefit of PRP procedures in ACLR.

## Supporting information


**Appendix S1** Checklist of the PRISMA for systematic review and meta‐analysis.

**Appendix S2** MIBO checklist for clinical studies evaluating PRP.

**Appendix S3** Searching strategies used for initial retrieval in the databases.

**Appendix S4** Summary of funding source of the included studies.
