# Supplementary material for: Effectiveness of Platelet‐Rich Plasma in Anterior Cruciate Ligament Reconstruction: A Systematic Review of Randomized Controlled Trials
Source: Orthop Surg. 2022 Sep 2;14(10):2406–17. doi: 10.1111/os.13279 (PMC9531067; doi:10.1111/os.13279)
Supplement: Supplementary file 2 — Appendix S2 MIBO checklist for clinical studies evaluating PRP. [file OS-14-2406-s004.doc]

| **Appendix 2. MIBO checklist for clinical studies evaluating PRP** | | | |
| --- | --- | --- | --- |
| **Section / Topic** | **Item**  **number** | Checklist item | Reported on  Page No |
| Study design | 1 | Study conducted in accordance with CONSORT (RCT), STROBE (cohort, case-control or cross-sectional) or PRISMA (meta-analysis) guidelines | 6 |
|  | 2 | Relevant institutional and ethical approval | Not needed |
| Recipient details | 3 | Recipient demographics (including age and gender) | 8-9 |
|  | 4 | Comorbidities (including underlying diabetes, blood dyscrasia, inflammatory conditions, pre-existing joint pathology and smoking status) | Not available |
|  | 5 | Current anti-inflammatory or anti-platelet medications | Not available |
| Injury details | 6 | Diagnosis (including relevant grading system and chronicity) | 6 |
|  | 7 | Results of any pre-operative imaging | Not needed |
|  | 8 | Previous surgical or biological treatments for current injury | Not needed |
| Intervention | 9 | Intervention described sufficiently to enable replication | 9-10 |
|  | 10 | Operative findings | Not available |
| Whole blood processing | 11 | Whole blood storage environment (including concentration and volume of anticoagulant, temperature and light exposure) | 10 (Table 2) |
| Whole blood characteristics | 12 | Whole blood platelet, differential leukocyte and red cell analysis of all samples | 10 (Table 2) |
| PRP processing | 13 | PRP processing described sufficiently to enable replication (including commercial kit details and Spin protocol) | 10 (Table 2) |
|  | 14 | Platelet recovery rate of protocol | Not available |
|  | 15 | PRP storage temperature and light exposure | Not available |
|  | 16 | Time between blood drawing, PRP processing, activation and delivery | 10 (Table 2) |
| PRP characteristics | 17 | PRP format (for example liquid, gel, membrane) | 10 (Table 2) |
|  | 18 | PRP platelet, differential leukocyte and red cell analysis of all samples | 10 (Table 2) |
| Activation | 19 | Activation described sufficiently to enable replication (including volume and concentration of activating agent) | 10 (Table 2) |
| Delivery | 20 | Point of delivery (intraoperative and/or postoperative or serial) | 9 (Table 1) |
|  | 21 | PRP delivery described sufficiently to enable replication (including volume delivered, concomitant use of stem cells or cytokines, and  details of carrier or scaffold) | 9 (Table 1) |
| Post-operative care | 22 | Rehabilitation protocol sufficiently described to enable replication (including immobilization and physical therapy) | 9 (Table 1) |
| Outcome | 23 | Outcome assessments include functional outcomes and recording of complications (including infection and need for further surgery). If  performed radiographic outcomes, physical examination findings, return to activities and satisfaction. | 10-12 |
