# Supplementary material for: Effectiveness of Platelet‐Rich Plasma in Anterior Cruciate Ligament Reconstruction: A Systematic Review of Randomized Controlled Trials
Source: Orthop Surg. 2022 Sep 2;14(10):2406–17. doi: 10.1111/os.13279 (PMC9531067; doi:10.1111/os.13279)
Supplement: Supplementary file 3 — Appendix S3 Searching strategies used for initial retrieval in the databases. [file OS-14-2406-s001.doc]

**Appendix 3. Searching strategies used for initial retrieval in the databases**

1. **PubMed**

**#1** (Anterior Cruciate Ligament[MeSH]) OR (Anterior Cranial Cruciate Ligament) OR (Cranial Cruciate Ligament) OR (Cranial Cruciate Ligaments) OR (Cruciate Ligament, Cranial) OR (Cruciate Ligaments, Cranial) OR (Ligament, Cranial Cruciate) OR (Ligaments, Cranial Cruciate) OR (Cruciate Ligament, Anterior) OR (Anterior Cruciate Ligaments) OR (Cruciate Ligaments, Anterior) OR (Ligament, Anterior Cruciate) OR (Ligaments, Anterior Cruciate) OR (Anterior Cruciate Ligament Reconstruction) OR (ACL)

**#2** (Reconstructive Surgical Procedures[MeSH]) OR (Procedure, Reconstructive Surgical) OR (Procedures, Reconstructive Surgical) OR (Surgical Procedure, Reconstructive) OR (Surgical Procedures, Reconstructive) OR (Reconstructive Surgery) OR (Reconstructive Surgeries) OR (Surgeries, Reconstructive) OR (Surgery, Reconstructive) OR (Reconstructive Surgical Procedure) OR (Reconstruction)

**#3** #1 AND #2

**#4** (Platelet-Rich Plasma [MeSH]) OR (Plasma, Platelet-Rich) OR (Platelet Rich Plasma) OR (PRP)

**#5** (Platelet-Rich Fibrin [MeSH]) OR (Fibrin, Platelet-Rich) OR (Platelet Rich Fibrin) OR (L-PRF) OR (Leukocyte- and Platelet-Rich Fibrin) OR (Leukocyte and Platelet Rich Fibrin) OR (PRF)

**#6** #4 OR #5

**#7** #3 AND #6

1. **Embase**

**#1** 'anterior cruciate ligament'/exp OR 'anterior cranial cruciate ligament' OR 'cranial cruciate ligament' OR 'cranial cruciate ligaments' OR 'cruciate ligament, cranial' OR 'cruciate ligaments, cranial' OR 'ligament, cranial cruciate' OR 'ligaments, cranial cruciate' OR 'cruciate ligament, anterior' OR 'anterior cruciate ligaments' OR 'cruciate ligaments, anterior' OR 'ligament, anterior cruciate' OR 'ligaments, anterior cruciate' OR 'anterior cruciate ligament reconstruction' OR 'acl'

**#2** 'platelet-rich plasma cell'/exp OR 'plasma, platelet rich' OR 'platelet rich plasma' OR 'prp' OR 'platelet rich fibrin' OR 'fibrin, platelet rich' OR 'platelet rich fibrin' OR 'l prf' OR 'leukocyte-platelet rich fibrin' OR 'leukocyte platelet rich fibrin' OR 'prf'

**#3** #1 AND #2

1. **CENTRAL**

**#1** MeSH descriptor: [Anterior Cruciate Ligament] explode all trees

**#2** (Anterior Cranial Cruciate Ligament) OR (Cranial Cruciate Ligament) OR (Cranial Cruciate Ligaments) OR (Cruciate Ligament, Cranial) OR (Cruciate Ligaments, Cranial) OR (Ligament, Cranial Cruciate) OR (Ligaments, Cranial Cruciate) OR (Cruciate Ligament, Anterior) OR (Anterior Cruciate Ligaments) OR (Cruciate Ligaments, Anterior) OR (Ligament, Anterior Cruciate) OR (Ligaments, Anterior Cruciate) OR (Anterior Cruciate Ligament Reconstruction) OR (ACL)

**#3** #1 OR #2

**#4** MeSH descriptor: [Platelet-Rich Plasma] explode all trees

**#5** (Plasma, Platelet-Rich) OR (Platelet Rich Plasma) OR (PRP) OR (Platelet-Rich Fibrin) OR (Fibrin, Platelet-Rich) OR (Platelet Rich Fibrin) OR (L-PRF) OR (Leukocyte- and Platelet-Rich Fibrin) OR (Leukocyte and Platelet Rich Fibrin) OR (PRF)

**#6** #4 OR #5

**#7** #3 AND #6
