# Supplementary material for: Effectiveness of Platelet‐Rich Plasma in Anterior Cruciate Ligament Reconstruction: A Systematic Review of Randomized Controlled Trials
Source: Orthop Surg. 2022 Sep 2;14(10):2406–17. doi: 10.1111/os.13279 (PMC9531067; doi:10.1111/os.13279)
Supplement: Supplementary file 4 — Appendix S4 Summary of funding source of the included studies. [file OS-14-2406-s002.doc]

| **Appendix 4. Summary of funding source of the included studies** | |
| --- | --- |
| **Author/year** | **Funding source** |
| Silva, 2009^20^ | Not available |
| Cervellin, 2012^21^ | Partially supported by the Italian Ministry of Health |
| Azcárate, 2014^22^ | Not available |
| Vogrin, 2010^23^ | Not available |
| Orrego, 2008^24^ | Supported by the Universidad de Los Andes, Santiago, Chile |
| Rupreht, 2012^25^ | Not available |
| Nin, 2009^14^ | Not available |
| Seijas, 2013^26^ | Not available |
| de Almeida, 2012^27^ | Criogenesis and Haemonetics Corp provided the personnel support and platelet-rich plasma separation system used in this study |
| Vadala, 2013^28^ | Not available |
| Vogrin, 2010^29^ | No funding |
| Mirzatolooei, 2013^30^ | No funding |
| Walters, 2018^31^ | B.B.B. is a consultant for Arthrex Inc. B.L.W. has received education and hospitality funds from Arthrex Inc, Smith & Nephew, and Zimmer Biomet; hospitality from Microport Orthopedics Inc; and royalties from Arthrex Inc. S.J.N. reports royalties, consulting fees, and hospitality from Arthrex Inc. |
| Seijas, 2013^32^ | Not available |
| Rupreht, 2013^33^ | Not available |
| Starantzis, 2014^34^ | the 2nd Department of Radiology, University of Athens |
